# Not Up for Discussion: Applying Lukes’ Power Model to the Study of Health System Corruption: Comment on "We Need to Talk About Corruption in Health Systems"

**DOI:** 10.15171/ijhpm.2019.75

**Published:** 2019-09-29

**Authors:** Lucy Reynolds

**Affiliations:** London School of Hygiene and Tropical Medicine, London, UK.

**Keywords:** Corruption, Power, Privatisation, Censorship, Whistleblowing

## Abstract

This companion paper suggests the potential benefits of applying Steven Lukes’ dimensions of power model to the study of corruption in health systems. Lukes’ model sets out three "faces of power" classified by their influence on political discourse, resulting in overt, covert and latent discussion of issues depending on the degree of their alignment with the agenda of dominant power interests.

His concept that differential access to public discourse varies according to this alignment implies the potential for identifying more serious forms of corruption by the mismatch between their practical importance and the amount of open debate addressing them. These two variables are in practice inversely related, and do not, as might be expected, correlate, with more important topics receiving more public attention. Lukes’ model would predict and can explain such inversion of public priorities, which tells us that observed suppression of public debate might efficiently direct the interest of researchers and the efforts of those seeking to further the public good on to the key issues needing discussion and resolution.

The commentary goes on to examine whether the most serious and dangerous forms of corruption might therefore also be the most invisible, and suggests that whistleblower reports should be considered a key data source for research into high-level corruption in health systems, including redirection of policy decisions away from those which are in the public interest.

The accompanying paper^1^ on the topic of health system corruption, highlighting a striking knowledge gap and addressing multiple aspects of this criminality with a variety of practical examples. This present commentary will argue the centrality of the power dimension in dealing intelligently with corruption as a phenomenon.

Hutchinson, Balabanova, and McKee apply Kingdon’s policy streams model,^2^ and the Zyglidopoulos^3^ research framework which categorises by individuals, organizations and industries, countries, and cultural contexts. These are ways to classify incidents of corruption which can aid researchers in structuring their observations, but neither provides conceptual illumination of how corruption enters a system, perpetuates and expands itself, and defends against its own exposure in order to safeguard gains to its sponsors and organisers.

Lukes advances a framework for “the dimensions of power,”^4^ relating the exercise of power to the representation of issues in the public eye. He postulates an “overt” level 1, in which issues can be discussed openly in the public domain, a “covert” level 2, in which debate is hard to start and tends to exhibit major skews and blind spots, and finally a “latent” level 3, covering issues which remain permanently off the public agenda because their discussion threatens powerful interests. Lukes sought in this work to develop the previously dominant paradigm which opposed levels 1 and 2, the themes of public debate approved by the power source (which reach the “overt” agenda) versus the agendas raised by other interested groups which are excluded from or misrepresented in media discussion (limited to “covert” status by exclusion from the mainstream discourse). This exclusion results in underrepresentation of important problems on the public agenda. He wished to focus attention on further complexity in the situation, with the existence of a less detectable level of exercise of power acting to keep some important issues at the “latent” undiscussed level, so that the most contentious issues of all cannot be addressed and appropriate solutions formulated. Multiple mechanisms of action might contribute to achieving and maintaining latency of some memes, for example the deterrent effect of punishment and public opprobrium for those raising the issues, or the lack of developed conceptual frameworks to support the debate so that some forms of dissatisfaction remain too hard to formulate in a coherent fashion at all, let alone to communicate successfully to others as a prelude to demanding public attention to the matter.

Hutchinson et al note:

“*A view in some quarters that concerns about corruption divert attention from more important issues…. Some of those adopting this paradigm see a focus on corruption as a manifestation of the neoliberal attack on the state, noting how it was prioritized by development agencies in the 1980s during the Reagan-Thatcher era, when many public health systems were being dismantled.”* ^1^

Lukes’ three-level framing implies that the more a type of corruption is discussed, the less significant it is. Here we observe that a focus on petty corruption by state officials appears to have been used by the neoliberal vanguard to provide distraction during the inception of a different form of corruption at much higher level: not pocketing small cash sums, but wholesale diversion of public budgets into the hands of large-scale commercial interests through national privatisation plans.

Evidence shows that falling performance in national health outcomes accompanies advancing privatisation.^5^ For example, the following graph (see Figure) plots the cost of healthcare systems in high and middle-income countries against the lifespan of the populations they serve, using the the international dataset compiled by the Organisation for Economic Co-operation and Development (OECD). It shows how additional investment affects the health of the population differently in national public monopoly healthcare systems and in marketized and privatised healthcare systems.

**Figure:**
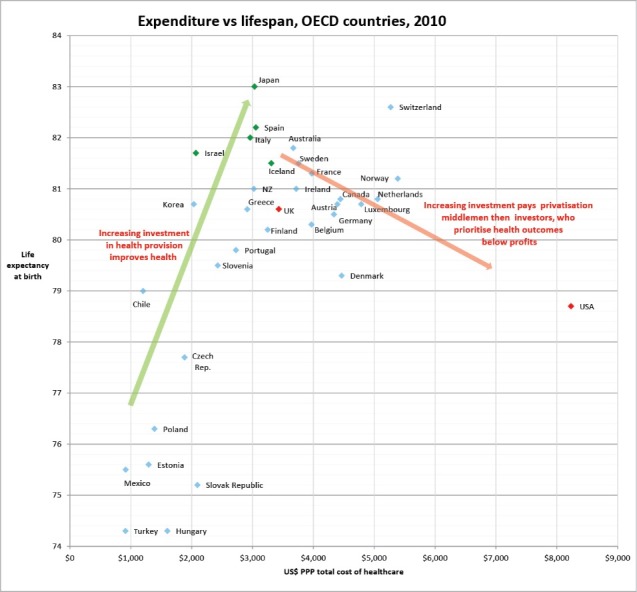
Cost Per Person of National Healthcare Systems Versus National Lifespan. Abbreviation: OECD, Organisation for Economic Co-operation and Development.

We can see that for publicly funded healthcare systems, which are distributed around the green arrow to the left of the graph, putting in higher state funding tends to be statistically associated with higher lifespan in the general population. We can also see that these systems present excellent value for money in terms of the population level lifespans achieved.

In contrast, countries with more expensive national healthcare systems health systems (those from the centre to the right side of the graph) are those which have commenced the transition to a highly marketized model of care as exemplified by the US system (on the far right), which despite spending over $8000 per year per head of population, is associated with life expectancy lower than that of any other OECD country spending above $2200 per capita. In this group around the red arrow, the higher the per capita state funding, the poorer the outcome at population level.

It would be fair to comment that such observational large-scale data sets may be vulnerable to confounding by unexplored factors, so the argument is strengthened by the fact that a plot of infant mortality data presents a very similar picture, though the confounders will be largely different. Here too, it can be observed that extra investment into non-marketised public healthcare improves infant survival across the whole population, but extra investment into marketized and marketising public healthcare systems seems to reduce their success in terms of reducing infant mortality.

There are multiple contributors to this apparently perverse result on the right-hand side of the graph. Critics of privatisation of national health systems usually focus on exclusion from care, and indeed we do see health outcomes at population level in such systems depressed by the exclusion of a substantial proportion of the population from access to needed medical care. However, fewer are aware that for-profit health facilities tend to encourage overtreatment of affluent patients, which can also be harmful to health. In this case the extra money spent achieves no gains for the patients, rather harm to their health motivated by the opportunity to increase profits by creating patient demand for medically inappropriate care.

For those that are not excluded from care, changes from marketisation both incentivise and enable diversion of scarce public funds away from provision of effective healthcare and toward other applications, such as advertising budgets, replication of administrative functions in competing organisation, and dividends. Furthermore the running of a market within a national healthcare system to enable private provision itself absorbs a substantial proportion of a national health budget. Meanwhile the chance to make profits incentivises profit-related overtreatment, for instance as reported by the US Department of Justice, which has reached over seventy legal settlements with hospitals involved in implanting cardiac devices without medical justification in order to increase the income to these hospitals from the state Medicare scheme.^6^ Such behaviour not only wastes scarce funding but reduces healthcare performance at a population level. Thus the poor outcomes from marketized health systems result from a combination of complete or partial exclusion for some with the harms from profit-driven overtreatment for others.

Should healthcare privatisation ushered in under neoliberal arguments not thus be itself conceptualised as a form of active corruption? There is some evidence that the corrupting elements themselves do view privatisation that way. John Perkins, formerly employed as a marketer of privatisation schemes to African, Asian and South American governments, explicitly classifies the international neoliberal attack fronted by the World Bank, and IMF (the USA’s “Reaganomics” and Latin America’s “Thatcherismo”^7^) as corrupt and corrupting in his account of his own role “Confessions of an Economic Hit Man.”^8^ Likewise, in the interview of former IMF Chief Economist Joseph Stiglitz with investigative journalist Greg Palast, Stiglitz labels one of privatisation’s standard steps “briberization.”^9^

Petty corruption, such as under-the-counter personal payments to clinicians, is at the first “overt” level: it can be discussed explicitly and honestly, and indeed most of the discussion of corruption in healthcare focuses on these relatively trivial and inexpensive misbehaviours. In contrast, corruption at the highest level, as documented by Perkins and Palast, operates at Lukes’ “latent” level 3, where public discussion of the problem is rare despite its gravity, but the budgets raided can run into hundreds of billions of pounds. This blindness of the public discourse to all but trivial corruption squares with the striking lack of funding earmarked to research systemic corruption. In a rational and honest system, finding and cleaning up corruption would be prioritised, facilitated, and lavishly funded, because such savings have only benefits for service quality.

Lukes’ concept can usefully be reverse-engineered: if researchers raise an issue of corruption and find that it is promptly addressed and rationally resolved, it is a level 1 issue, on the overt agenda. A level 2 issue is discussed but reframed; it may be addressed but never in a way likely to resolve it. Important issues which commentators find impossible to have publicly discussed at all despite their evident significance and urgency are thereby flagged up as level 3, held at the latent level by powerful vested interests. An example might be the lack of effective system-level action to block sales inducements to prescribers, as identified by Hutchison et al^1^: an organisational blind eye turned to such practices might suggest systemic corruption. Another is the minimal research attention so far paid to the fundamental professional conflict of interests of the insurance industry man heading National Health Service (NHS) England.^10^ A further sign attesting to behind-the-scenes suppression of open debate is the recent academic censorship debacle at the top of the Cochrane Collaboration.^11^

Perkins explains the sources and methods of the neoliberal infiltration from the perspective of one of its front-line salesmen. His search for a publisher demonstrated why and how the worst corruption stays hidden at the latent level, away from public debate:

“*In 2003, the president of a major publishing house that is owned by a powerful international corporation read a draft of what had now become Confessions of an Economic Hit Man. He described it as ‘a riveting story that needs to be told.’ Then he smiled sadly, shook his head, and told me that since the executives at world headquarters might object, he could not afford to risk publishing it.”* ^6^

Whistleblowers are a common consequence of hidden corruption, and their reports are among the best data sources concerning malfeasance at systems level. Doctors Holt,^12^ Alexander, and Drew^13^ have written on the attempted silencing of clinicians who speak out for the protection of their patients:

“*Francis found that the problem was widespread and systemic within the NHS: “I heard shocking accounts of the way some people have been treated when they have been brave enough to speak up … The number of people who wrote to the review who reported victimisation or fear of speaking up has no place in a well-run, humane and patient centred service.” He also found that the law is weak and does not protect whistleblowers, something campaigners have been reporting for some time”* (Holt).

“…*Raj Mattu, a whistleblowing cardiologist. An employment tribunal ruled last year that Mattu was ‘blameless’ and unfairly sacked after voicing concerns about patient safety. An astonishing campaign was waged against Mattu: ‘Soon, the single complaint against Mattu had become 35, then 200, ranging from questions over his qualifications to charges of serious criminal conduct outside work. These were sent to the GMC, CQC, the Strategic Health Authority and three different police forces; by 2009, all had been investigated and found to be false’”* (Alexander and Drew).

If Lukes model does reflect the structuring of public discourse, then, logically, more attention should be paid to less-publicised aspects of public policy, especially to reading whistleblower reports and to protecting their authors: these public-spirited individuals shed rare spotlights on matters which we cannot safely leave to be conducted in the shade. If solutions to the worst problems are not to be kept permanently from us, we must study them by paying attention to any whistleblowing from Lukes’ third level, in conflict with dominant power agendas and not for public discussion. Whistleblower reports may be the only chance we have to find out about frightening problems such as the risks to intensive care patients from chronic and extreme understaffing of available doctors, as raised by Dr. Christopher Day.^14^ Perhaps those studying public health agendas should pay special attention to this particular data source: is not that which is intentionally concealed bound to be also that which is likely to be of greatest interest?

Whistleblower disclosures could be used to monitor and change the system when needed, for example through legislation to prevent ‘legal’ corruption as is the instance of induced prescribing and handing contracts to private providers while there is a conflict of interest between them and policy-makers. Occasionally such conflicts of interest do reach public attention, for instance those reported of Health Minister Matt Hancock in respect of Babylon’s GP-destabilising siphoning phone app,^15^ and of many of the House of Lords,^16^ but the discussion of such ideas is usually likely to have problems rising from the Level 2 agenda to Level 1, public debate, and this will require concerned people to promote public debate and discussion very actively. The Hutchinson et al paper is a welcome step in raising public attention to the problems of corruption in healthcare. It is to be hoped that this present commentary can encourage the adoption of Lukes’ three level framework to inform and structure future public debate on critical health issues, by focusing our attention not on the mainstream of public debate but instead directing it to those most-crucial matters which have been kept outside public awareness.

## Ethical issues

Not applicable.

## Competing interests

Author declares that she has no competing interests.

## Author’s contribution

LR is the single author of the paper.
